# Breathe Better After COVID: The Impact of a Two-Week Pulmonary Rehabilitation Program on Pulmonary Function, Inflammatory Markers, and Quality of Life in Post-COVID Syndrome

**DOI:** 10.3390/jcm14134533

**Published:** 2025-06-26

**Authors:** Monika Bal-Bocheńska, Justyna Wyszyńska, Magdalena Kołodziej

**Affiliations:** 1 Faculty of Health and Psychology Sciences, University of Rzeszów, al. Tadeusza Rejtana 16c, 35-959 Rzeszów, Poland; mbal@ur.edu.pl (M.B.-B.); jwyszynska@ur.edu.pl (J.W.); 2 Podkarpackie Center for Lung Diseases, University Clinical Hospital in Rzeszów, ul. Lubelska 2, 35-241 Rzeszów, Poland; 3 Faculty of Medicine, University of Rzeszów, al. Tadeusza Rejtana 16c, 35-959 Rzeszów, Poland

**Keywords:** post-COVID syndrome, pulmonary rehabilitation, pulmonary function, quality of life, inflammation, spirometry

## Abstract

**Background**: Post-COVID syndrome is characterized by persistent symptoms such as dyspnea, fatigue, and reduced exercise tolerance, which can significantly impair pulmonary function and quality of life. Pulmonary rehabilitation has been proposed as a potential intervention to address these challenges. This study aimed to evaluate the effects of a pulmonary rehabilitation program on pulmonary function, inflammatory markers, and quality of life in patients with post-COVID syndrome. **Methods**: A prospective, interventional, non-randomized clinical trial was conducted involving 77 participants (mean age 59.4 ± 11.6 years; 39% female) who attended a post-COVID care clinic in Rzeszów, Poland. The intervention included supervised respiratory and aerobic exercises, muscle strengthening, and body balance therapy, alongside motivational breathing therapy. Pulmonary function (spirometry, plethysmography, gasometry), inflammatory markers (CRP, WBC, D-dimer), and quality of life (WHOQOL-BREF) were assessed pre- and post-intervention. **Results**: Significant improvements were observed in pulmonary function parameters post-rehabilitation, including increases in forced vital capacity (FVC, 75% to 78.4%, *p* < 0.001), forced expiratory volume in one second (FEV1, 78.2% to 80.5%, *p* < 0.001), and total lung capacity (TLC, 67.3% to 71%, *p* < 0.001). The diffusing capacity for carbon monoxide (DLCO) improved by 6.2% (*p* < 0.001). Arterial oxygen pressure (PaO_2_) increased by 7.6 mmHg (*p* < 0.001). Markers of inflammation, including CRP (8.9 to 1.3 mg/dL, *p* < 0.001) and d-dimer (1722.2 to 203.4 ng/mL, *p* < 0.001), showed significant reductions. Quality of life improved across physical, psychological, and environmental domains. Sex, BMI, and baseline inflammatory markers were significant determinants of rehabilitation outcomes. **Conclusions**: A pulmonary rehabilitation program significantly improved pulmonary function, reduced systemic inflammation, and enhanced quality of life in individuals with post-COVID syndrome. The findings highlight the importance of tailored rehabilitation in mitigating long-term post-COVID sequelae. Future research should explore the long-term effects of rehabilitation and its applicability in diverse populations.

## 1. Introduction

The novel coronavirus, SARS-CoV-2, first identified in 2019, triggered a global pandemic with profound health implications. While the acute phase of COVID-19 has been extensively studied, a significant subset of patients experience persistent symptoms beyond the initial infection period, a condition termed post-COVID syndrome or long COVID. This syndrome includes a diverse range of symptoms, including dyspnea, fatigue, reduced exercise tolerance, cognitive disturbances, and psychological distress, which can persist for months after the acute illness [1].

Pulmonary rehabilitation is a well-recognized approach for managing chronic respiratory conditions, particularly chronic obstructive pulmonary disease. Pulmonary rehabilitation programs typically involve a combination of exercise training, education, and psychosocial support, with the goal of enhancing both physical and mental health [2]. Given the respiratory complications associated with post-COVID syndrome, pulmonary rehabilitation has been proposed as a potential therapeutic strategy to alleviate persistent symptoms and enhance quality of life in affected individuals.

Recent systematic reviews and meta-analyses have assessed the efficacy of pulmonary rehabilitation in patients with post-COVID syndrome. A comprehensive review by Martínez-Pozas et al. analyzed data from 16 studies involving 1027 patients and found that pulmonary rehabilitation significantly improved dyspnea, physical function, quality of life (both global and physical domain), anxiety, and depression. However, the effect on fatigue was not significant [3]. Another meta-analysis conducted by Meléndez-Oliva et al., which included 34 studies involving 1970 patients, revealed that pulmonary rehabilitation had moderate to significant positive effects on dyspnea, physical function, quality of life, and depressive symptoms when compared to standard care intervention. No significant differences were found in fatigue or anxiety levels after pulmonary rehabilitation intervention [4].

The beneficial effects of pulmonary rehabilitation in post-COVID patients are achieved through multiple mechanisms. Exercise training within these programs improves cardiovascular fitness and muscular strength, helping to counteract the deconditioning caused by prolonged illness or inactivity. Breathing exercises and respiratory muscle training enhance ventilatory efficiency and alleviate dyspnea. Furthermore, the educational and psychosocial aspects of pulmonary rehabilitation help to manage anxiety, depression, and other psychological challenges frequently observed in individuals with post-COVID syndrome [5,6].

Despite the promising findings, there is variability in the design and implementation of pulmonary rehabilitation programs for post-COVID patients. Factors such as the mode of delivery (face-to-face vs. telerehabilitation), duration, intensity, and specific components of the intervention can influence outcomes. A study comparing face-to-face pulmonary rehabilitation and telerehabilitation found that both approaches effectively improved health status in patients with lung diseases, including COVID-19. However, face-to-face pulmonary rehabilitation showed superior improvements in the physical domain of quality of life [7].

While the beneficial effects of pulmonary rehabilitation in post-COVID patients have been documented, the majority of existing studies have focused on outpatient or telerehabilitation programs delivered over longer durations. In contrast, there is limited evidence evaluating short-term, inpatient rehabilitation protocols and their multidimensional outcomes—particularly in hospitalized individuals recovering from COVID-19 [4]. Given the heterogeneity of post-COVID syndrome and the variability in treatment responses, further research is needed to refine and optimize rehabilitation strategies for this population. By examining a structured, two-week inpatient program and incorporating assessments of pulmonary function, inflammatory biomarkers, and quality of life, this study seeks to address these gaps. The aim of this study is to evaluate the effectiveness of an inpatient pulmonary rehabilitation program in improving respiratory function, reducing systemic inflammation, and enhancing quality of life in patients with post-COVID syndrome.

## 2. Materials and Methods

### 2.1. Study Design

This study was a prospective, interventional, non-randomized clinical trial conducted to evaluate the effects of pulmonary rehabilitation on pulmonary function and quality of life in patients with post-COVID syndrome. The trial was registered with ClinicalTrials.gov (ID: NCT04751617) and adhered to the STROBE guidelines for observational studies.

### 2.2. Participants

This study recruited seventy-seven participants. The recruitment period spanned from January 2021 to March 2023, and the participants were recruited from the post-COVID care clinics at Provincial Clinical Hospital No. 1 in Rzeszów and Podkarpackie Center for Lung Diseases in Rzeszów.

Eligible participants for this study were adults aged 18 years or older who had recovered from laboratory-confirmed COVID-19 (via RT-PCR or antigen testing) and continued to experience persistent symptoms such as dyspnea, fatigue, or reduced exercise tolerance for at least 12 weeks post-infection. Recovery was defined as the resolution of acute infection and negative follow-up testing or clinical clearance. All participants had to be medically stable, functionally independent in daily activities, and referred for post-COVID care by a specialist.

**Figure 1:**
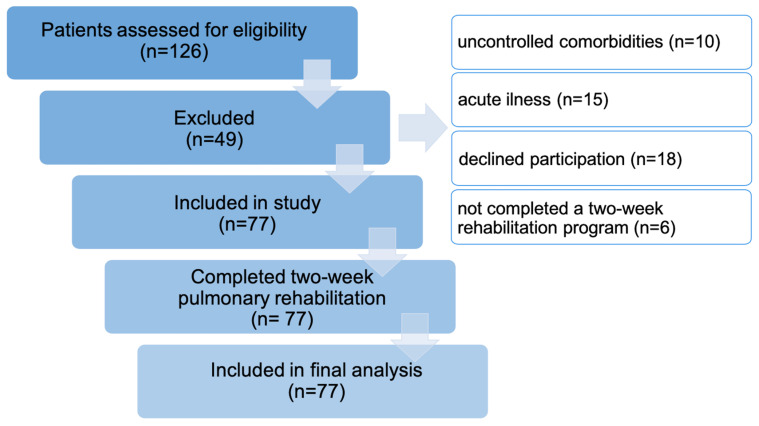
Flow diagram of participants screening, inclusion, and completion of the pulmonary rehabilitation program.

Exclusion criteria included the following: current or recent (within 3 months) acute illness or infection; uncontrolled chronic diseases (e.g., unstable cardiovascular conditions, decompensated diabetes, or severe renal/hepatic dysfunction); severe neurocognitive impairment or psychiatric illness precluding participation; participation in another clinical trial; any contraindication to physical activity or pulmonary rehabilitation. The participant selection process is illustrated in Figure 1.

### 2.3. Intervention

The pulmonary rehabilitation program began with early in-bed mobilization exercises, focusing on frequent posture changes to promote lung function and overall mobility. This was followed by supervised exercise training, diaphragmatic breathing and respiratory muscle training, as well as muscle strengthening exercises. In addition to standard breathing exercises, patients performed exercises with an incentive spirometer to enhance respiratory muscle activation and lung expansion. This device-based training was included as part of the daily respiratory therapy regimen to support ventilatory efficiency and improve inspiratory capacity. Additionally, participants were engaged in aerobic exercises such as walking, stationary bike use, and climbing stairs, aimed at improving cardiovascular endurance and overall physical conditioning. Body balance therapy was also incorporated to enhance stability and coordination. The standard patient education provided during the rehabilitation program included the role of physical activity, respiratory hygiene, and smoking cessation for participants who reported current or past smoking, as part of general health promotion efforts.

The duration of the program was fixed at 14 days for all participants. Each participant completed a structured sequence of 12 rehabilitation sessions, delivered six times a week over two weeks. Each session lasted approximately 45 min.

In addition to the supervised sessions, every patient was advised to engage in independent motivational breathing therapy every two hours, seven days a week, to continually promote respiratory health and function.

While the core structure of the rehabilitation program was standardized for all participants, selected components were personalized. Adjustments in exercise intensity and progression were based on baseline clinical indicators such as pulmonary function and individual physical tolerance, as assessed by the supervising physiotherapist. These adaptations were designed to ensure safety and optimize the rehabilitation response for each patient, without deviating from the overall protocol framework.

### 2.4. Outcome Measures

#### 2.4.1. Primary Outcomes

The primary outcomes were changes in pulmonary function assessed by spirometry, plethysmography, lung diffusion test, and gasometry.

#### 2.4.2. Spirometry

Parameters measured in spirometry were forced vital capacity (FVC), forced expiratory volume in one second (FEV1), FEV1/FVC ratio, peak expiratory flow (PEF), and maximum expiratory flow in 75, 50, and 25% (MEF75%, MEF50%, MEF25%). Spirometry was performed according to standard protocols [8], using a Pneumo abcMed ATS/ERS 2005 spirometer.

#### 2.4.3. Body Plethysmography

The body plethysmography parameter was the total lung capacity (TLC), and with the lung diffusion test, the diffusing capacity for carbon monoxide (DLCO) was assessed. All above parameters were expressed as a percentage of the normal values, estimated for each patient separately in the designed facility. The test was performed according to standard protocols [9], using MES LungTest 1000.

#### 2.4.4. Gasometry

Gasometry parameters were partial pressure of oxygen (PaO_2_) and partial pressure of carbon dioxide (PaCO_2_), taken from the capillary blood, and expressed in mmHg. Gasometry was performed according to standard criteria [10], using an ABL90 FLEX plus analyzer (Radiometer, Copenhagen, Denmark).

#### 2.4.5. Secondary Outcomes

Secondary outcomes included health-related quality of life (QoL) and blood parameters. QoL was estimated by the WHOQOL-BREF questionnaire, a widely used tool for assessing quality of life across four key domains: physical health, psychological well-being, social relationships, and environmental factors. It consists of 26 items, each scored on a 5-point Likert scale, with higher scores indicating better quality of life. Domain scores are calculated by summing item responses within each domain, then converting these to a 0–100 scale for easier interpretation and comparison [11]. Blood was examined to assess inflammation using the C-reactive protein (CRP) test. White blood cells (WBCs) were evaluated and the D-dimer test was used to assess the coagulation system. Blood examination was performed according to standard criteria [12,13].

All assessments were conducted at baseline (pre-intervention) and at the end of the rehabilitation intervention period (post-intervention).

### 2.5. Data Collection

Data were collected through in-person assessments at the hospital before and after the intervention. Baseline assessments were conducted on the first day of rehabilitation (day 0), prior to the initial session. Post-intervention measurements were performed on day 14, the final day of the rehabilitation program. Spirometry, plethysmography, lung diffusion, gasometry, and blood tests were conducted by experienced hospital personnel, following standard criteria. All tests were performed by the same medical personnel to ensure consistency and minimize variability. The intervention was performed by a physiotherapist, who was blind to the process of testing patients. Questionnaires before and after this study were administered by the same physiotherapist, who did not supervise patients during the interventional part of this study.

### 2.6. Statistical Analysis

Sample size calculation was performed based on expected changes in FVC, TLC, and QOL scores, with an alpha level of 0.05 and 80% power, resulting in a required sample size of minimum 39 participants. The data analysis was performed using SPSS software (v 29.0). The distribution of continuous variables was assessed using the Shapiro–Wilk test. As all assessed variables significantly deviated from normal distribution, non-parametric tests were applied for statistical comparisons. Categorical variables were summarized using percentages, whereas continuous variables were presented as mean and standard deviation. Between-group differences were analyzed using the Wilcoxon test. Mann–Whitney U and Spearman R tests and the linear regression model were used to assess the rehabilitation effectiveness. Statistical significance was considered as *p* < 0.05.

### 2.7. Ethical Considerations

This study was approved by the Bioethics Commission of the University of Rzeszów (Approval Number: 13/01/2021). Written informed consent was obtained from all participants prior to enrollment. This study adhered to the principles of the Declaration of Helsinki and Good Clinical Practice guidelines. Participants were assured of the confidentiality of their data and their right to withdraw from this study at any time without any impact on their medical care.

## 3. Results

### 3.1. Participants Characteristics

This study included 77 participants, including 39% females and 61% males (Table 1).

The average participant age was 59.4 ± 11.6 years. Most participants (63.6%) lived in urban areas, while 36.4% resided in rural settings. Educational backgrounds varied, with 42.9% completing secondary education, 35.1% higher education, 20.8% vocational education, and only 1.3% reporting primary education.

Occupationally, 61% of participants were engaged in physical labor, 28.6% in white collar work, and 10.4% performed both. Based on BMI classification, 16.9% were of normal weight, 53.2% were overweight, and 29.9% were obese, with a mean BMI of 28.76 ± 4.05 kg/m^2^. Smoking prevalence was 31.2% among regular smokers, 6.5% among occasional smokers, and 62.3% among nonsmokers. Smoking was significantly higher among males (38.3%) than females (20%, *p* < 0.05).

### 3.2. Pulmonary Function and Gas Exchange

Significant improvements were observed in volume pulmonary function parameters post-rehabilitation (Table 2).

**Figure 2:**
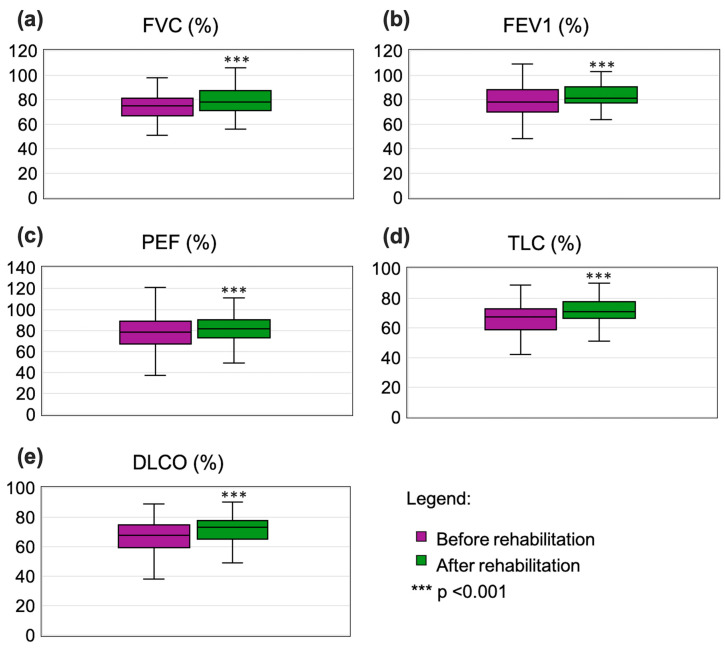
Box whisker plots showing comparison of results pre- and post-rehabilitation for the following parameters: (**a**) FVC, (**b**) FEV1, (**c**) PEF, (**d**) TLC, and (**e**) DLCO. Box represents median, 25th percentile, and 75th percentile, and whiskers represent min and max values.

The median FVC increased from 75% to 78.4% (*p* < 0.001), and FEV1 rose from 78.2% to 80.5% (*p* < 0.001) (Figure 2a,b). Similar to flow parameters, significant improvements were observed in PEF (78.7% to 81%; *p* < 0.001; Figure 2c) and all MEF values (Table 2). Males achieved significantly higher median FEV1 values than females (*p* < 0.001) (Supplementary Tables S1 and S2), with a negative correlation noted between FEV1 and age (R = −0.271, *p* < 0.05) (Supplementary Table S3).

While pre- and post-rehabilitation results indicated no signs of obstruction (FEV1/FVC > 75%), improvements in median TLC were significant (from 67.3% to 71%, *p* < 0.001; Figure 2d), with median DLCO values increasing from 67.8% to 74% (*p* < 0.001; Figure 2e).

**Figure 3:**
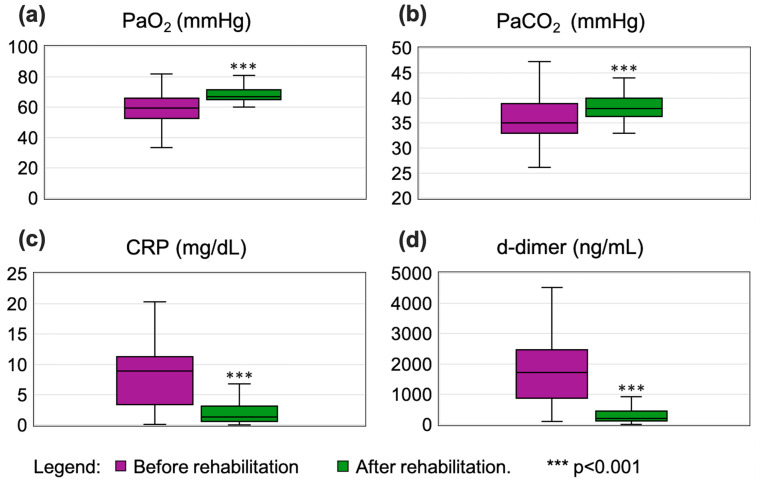
Box whisker plots showing comparison of results pre- and post-rehabilitation for the following parameters: (**a**) PaO_2_, (**b**) PaCO_2_, (**c**) CRP, and (**d**) d-dimer. Box represents median, 25th percentile, and 75th percentile, and whiskers represent min and max values.

Arterial blood gas parameters also significantly improved. The median of PaO_2_ increased from 59.4 mmHg to 67 mmHg (*p* < 0.001; Figure 3a), and PaCO_2_ rose from 35 mmHg to 38 mmHg (*p* < 0.001; Figure 3b).

### 3.3. Inflammatory Markers and Blood Parameters

Markers of inflammation demonstrated marked reductions (Table 2). CRP levels decreased nearly sevenfold (from 8.9 mg/dL to 1.3 mg/dL; *p* < 0.001; Figure 3c), while d-dimer values decreased eightfold (from 1722.2 ng/mL to 203.4 ng/mL; *p* < 0.001; Figure 3d).

Sex differences were evident in blood parameters. While men exhibited a statistically significant rise in white blood cell count (WBC) post-rehabilitation (from 9.44 to 12.06 × 10^3^/µL; *p* < 0.05), women showed no significant changes (*p* > 0.05) (Supplementary Table S1). A negative correlation was observed between BMI and WBC (R = −0.316, *p* < 0.01) (Supplementary Table S3).

### 3.4. Quality-of-Life Assessments

**Figure 4:**
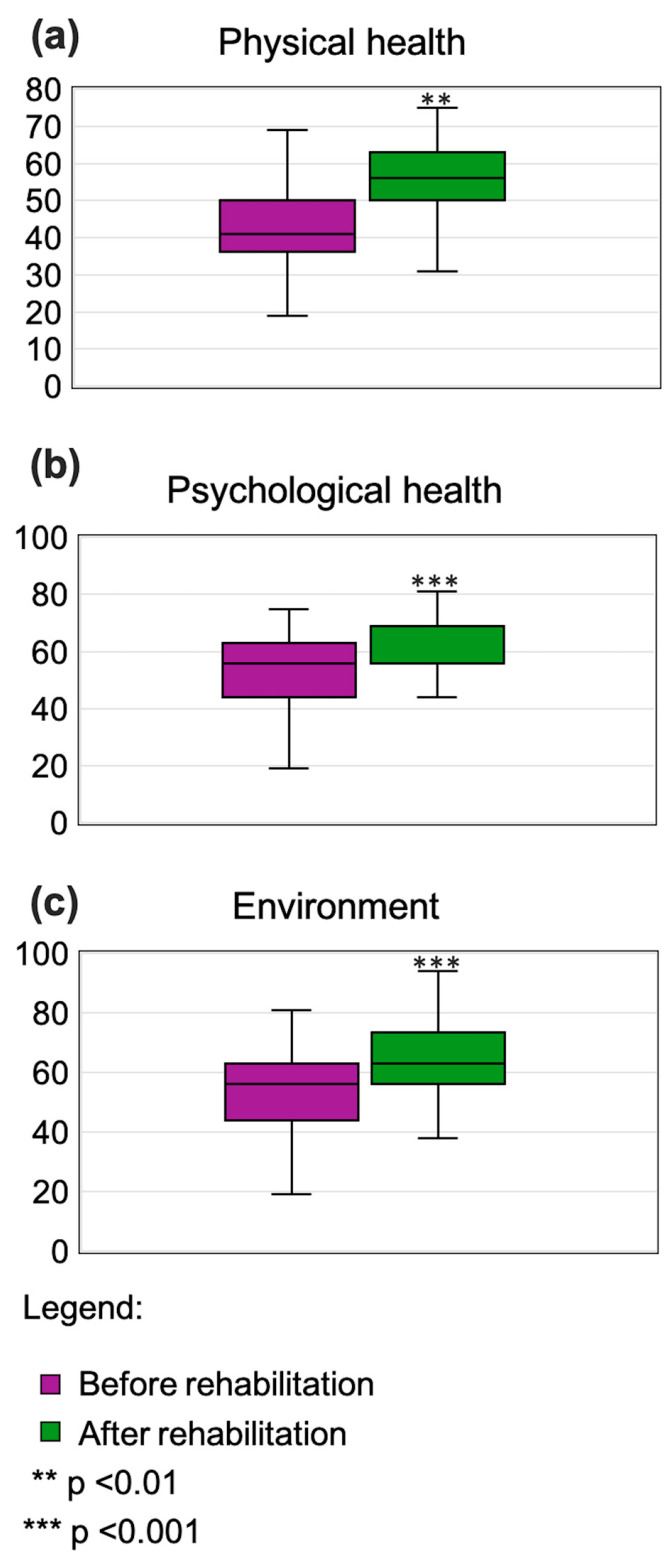
Box whisker plots showing comparison of results pre- and post-rehabilitation for parameters: (**a**) physical health, (**b**) psychological health, and (**c**) environment. Box represents median, 25th percentile, and 75th percentile, and whiskers represent min and max values.

Results of quality-of-life assessments are summarized in Table 2. Rehabilitation resulted in significant improvements in three of four quality-of-life domains: physical (*p* < 0.01; Figure 4a), psychological (*p* < 0.001; Figure 4b), and environmental (*p* < 0.001; Figure 4c).

### 3.5. Key Factors Influencing Rehabilitation Outcomes

Sex was a significant determinant of rehabilitation effects. Females showed greater improvements than males in psychological (16 vs. 0 points) and environmental (13 vs. 7 points) quality-of-life domains (*p* < 0.05), suggesting heightened sensitivity to psychosocial aspects of rehabilitation.

Inflammatory markers also played a role. Elevated baseline CRP correlated positively with social domain improvements, while higher d-dimer levels were associated with less progress. This indicates that systemic inflammation reduction supports social functioning, whereas coagulation-related factors may hinder it.

BMI was negatively correlated with WBC changes (R = −0.316, *p* < 0.01), suggesting that a higher BMI might reduce responsiveness to rehabilitation-induced immune adjustments. Additionally, age was correlated negatively with FEV1 improvement (R = −0.271, *p* < 0.05), highlighting the impact of age-related pulmonary changes on recovery potential. The above analysis is summarized in Table 3

## 4. Discussion

The findings of this study demonstrate that a structured pulmonary rehabilitation program is feasible and effective in enhancing respiratory function, exercise tolerance, and quality of life among patients experiencing persistent post-COVID-19 symptoms. To our knowledge, this is one of the few studies evaluating a comprehensive pulmonary rehabilitation program implemented in COVID-19 patients after the acute phase of the disease. A systematic review by Negrini et al. demonstrated that the current level of evidence regarding the effectiveness of pulmonary rehabilitation in COVID-19 patients is low, primarily due to the lack of studies evaluating specific interventions and the organization of rehabilitation services. In light of this gap, our findings provide valuable clinical data supporting the need for further research on the effectiveness of respiratory therapies [14]. Therapies based on respiratory muscle training, especially when combined and supervised, effectively improved quality of life and the strength and endurance of respiratory muscles in individuals with post-COVID syndrome, particularly in cases of pronounced dyspnea [15]. Implementing such programs in clinical practice may enhance patient motivation and therapeutic teams’ readiness to apply them in outpatient and hospital care.

In our study, post-COVID-19 patients underwent a supervised, multimodal pulmonary rehabilitation program consisting of six sessions per week, each lasting approximately 45 min. This protocol led to statistically significant improvements in FVC (from 75% to 78.4%), FEV1 (from 78.2% to 80.5%), and PEF (from 78.7% to 81%) of predicted values (*p* < 0.001 for all). These findings demonstrate the effectiveness of an intensive, structured rehabilitation program in enhancing both volume-related and flow-related pulmonary function.

When compared with previous studies, our results show consistent but broader improvements. Gloeckl et al. [6] evaluated a three-week inpatient pulmonary rehabilitation program and reported improvements in FVC of 7.7% and 11.3% in patients with mild/moderate and severe/critical COVID-19, respectively (*p* = 0.002 and *p* < 0.001), but did not observe significant changes in FEV1 or PEF. In contrast, our study demonstrated concurrent improvements in FVC, FEV1, and PEF, likely reflecting the higher intensity of our intervention.

The randomized controlled trial by Okan et al. [16], which implemented a five-week home-based telerehabilitation program with minimal supervision (one session per week), also reported improvements in FEV1, FVC, and MVV. While these findings align with ours, the magnitude of change was smaller, possibly due to a lower session frequency and reduced professional oversight. Additionally, our program included six supervised sessions per week, suggesting that both intensity and supervision may be key factors influencing the magnitude of the outcome.

In the study by Del Corral et al. [17], improvements were limited to PEF and observed only in the respiratory muscle training (RMT) group. No significant changes in FVC or FEV1 were reported. This contrasts with our findings and may be explained by the limited scope and lack of supervision in their intervention, which focused exclusively on RMT using a threshold pressure device.

Taken together, these comparisons underscore that improvements in pulmonary function following rehabilitation are closely tied to the intensity, duration, and comprehensiveness of the intervention. Our findings support the view that supervised, multimodal programs yield more consistent and robust benefits across multiple pulmonary parameters than isolated or less intensive models. Furthermore, in line with the definition of pulmonary rehabilitation provided by the American Thoracic Society and the European Respiratory Society [2], only interventions that integrate exercise training, education, and behavioral support should be classified as proper pulmonary rehabilitations. This distinction is essential when interpreting outcomes and comparing rehabilitation models across studies.

Although our study demonstrated statistically significant improvements in spirometric and functional parameters (e.g., FVC +3.4%, FEV1 +2.3%, DLCO +6.2%, PaO_2_ +7.6 mmHg), their clinical relevance must be critically evaluated in light of the minimal clinically important difference (MCID). The MCID represents the smallest change in a clinical outcome that patients perceive as beneficial, and that would justify a change in treatment. For FEV1, multiple authoritative sources suggest that an absolute change of at least 100 mL is generally considered clinically meaningful in populations with chronic respiratory diseases, such as chronic obstructive pulmonary disease (COPD) [18,19]. While our relative improvement in FEV1 was statistically significant, the corresponding absolute changes in many participants may have remained below this threshold, limiting the likelihood of noticeable improvements in symptoms such as dyspnea or exercise capacity. Similarly, for FVC, the MCID for percent predicted values in diseases such as idiopathic pulmonary fibrosis (IPF) is estimated to range from 2% to 6% [20]. In our study, the median percent predicted FVC increased from 75.0% to 78.4% (*p* < 0.001), approaching this clinically relevant range and suggesting a potential, though modest, functional benefit.

The improvements in DLCO and PaO_2_ observed after pulmonary rehabilitation are likely driven by better ventilation/perfusion (V/Q) matching—a key physiological adaptation to exercise training. Pulmonary rehabilitation enhances both cardiovascular performance and ventilatory efficiency, promoting the recruitment of additional alveolar units and pulmonary capillaries. These changes enhance the way air reaches the alveoli and how blood flows through the lungs, thereby making gas exchange more efficient.

In particular, increases in lung volumes such as TLC, FEV_1_, and FVC point to better lung mechanics and greater alveolar recruitment. The above leads to a more even distribution of ventilation relative to perfusion, helping to optimize the V/Q relationship. As a result, the coordination between airflow and blood flow improves, which can explain the observed increases in DLCO and PaO_2_ [21]. Although weight loss was not directly assessed in our study, it is acknowledged that even modest reductions in body weight, as demonstrated in bariatric surgery cohorts, can decrease pulmonary blood volume and reduce V/Q mismatch, thereby improving gas exchange and arterial oxygenation [22].

Few studies evaluate the effects of pulmonary rehabilitation programs in hospitalized patients. Hermann et al. assessed the impact of a pulmonary rehabilitation program by analyzing data from patients hospitalized due to COVID-19. A retrospective analysis of 28 COVID-19 patients revealed that post-COVID pulmonary rehabilitation improved physical performance and overall health status [23]. Similar patterns were observed in our study.

Restrictive pulmonary changes and a reduced diffusing capacity following lung injury contribute to long-term functional limitations and a decreased quality of life [6]. These factors may explain the persistent consequences observed in patients with COVID-19 and provide reasonable evidence supporting the need for rehabilitation in this patient population [24]. Although several years have passed since the onset of the pandemic, it remains unclear whether COVID-19 causes permanent lung damage, and if so, to what extent [25]. In our study, patients with a history of COVID-19 exhibited a restrictive pattern of pulmonary function, as assessed by TLC. Following the rehabilitation program, TLC improved by 3.7%. In the study by McDonald et al., pulmonary function impairment and increased lung fibrosis were observed in up to 62% of COVID-19 survivors [26]. Currently, there is emerging evidence suggesting a link between COVID-19 and the development of fibrotic lung disease, which represents a serious and long-term clinical concern [27].

In our study, patients demonstrated a reduced quality of life in both physical and psychological functioning prior to rehabilitation, which is consistent with the findings of Wong et al. [28]. After completing the rehabilitation program, a significant improvement was observed in three of four assessed quality-of-life domains: physical, psychological, and environmental. Similarly, Del Corral et al. reported an improved quality of life among patients with long-term post-COVID-19 symptoms following a home-based respiratory muscle training program; however, no improvements were noted in exercise tolerance or pulmonary function [17]. Although our study did not include direct measurements of exercise capacity, the results indicate a clear enhancement in self-perceived physical functioning, with scores rising from 41 to 56 points (*p* < 0.01), highlighting the effectiveness of a comprehensive rehabilitation program.

Sex appeared to influence rehabilitation outcomes, with women showing greater improvements than men in the psychological (16.0 vs. 0.0 points) and environmental (13.0 vs. 7.0 points) domains of quality of life. While previous studies have explored sex-related differences in post-COVID recovery [29], it has also been reported that women tend to experience higher levels of fatigue than men [30], which may partly explain their greater responsiveness to physiotherapy observed in our study. The observed improvements in WHOQOL-BREF domains—physical (+14.3 points), psychological (+6.7 points), and environmental (+9.2 points)—suggest meaningful perceived benefits in quality of life following rehabilitation. Although the WHOQOL-BREF lacks universally accepted MCID thresholds, distribution-based estimates provide useful benchmarks. For example, in patients with advanced lung cancer and mesothelioma, de Mol et al. reported MCIDs ranging from 0.88 to 1.55 points (0.5 SD) and 1.76 to 2.72 points (1 SEM) across domains [31]. All domain changes in our study substantially exceed these values, reinforcing the likelihood of clinical relevance. However, as these thresholds were derived from a different patient population, direct comparisons should be made cautiously. While our findings strongly suggest meaningful improvement, confirmation in post-COVID rehabilitation cohorts with anchor-based MCID estimates remains warranted.

Our study also underscores the need for further investigations into the role of serum CRP and D-dimer levels as potential prognostic markers during COVID-19. Findings by Ali et al. indicated that levels of D-dimers, CRP, and WBCs were significantly higher in patients who died from COVID-19 compared to those who survived [32]. In a multivariate analysis, elevated CRP concentrations were associated with an increased incidence of pneumonia. Based on these results, the authors suggest the necessity of prompt imaging diagnostics (e.g., computed tomography) and the implementation of more comprehensive therapeutic approaches, including both physiotherapy and psychological support [33]. The above is consistent with our findings, where CRP and D-dimer levels significantly decreased following the rehabilitation program, suggesting that comprehensive therapy may contribute to reducing systemic inflammation and potential complications.

In our study, median reductions in the inflammatory biomarkers CRP (−7.6 mg/dL) and D-dimer (−1.518.8 ng/mL) were substantial following rehabilitation. These changes align with existing evidence, suggesting that physical rehabilitation can attenuate systemic inflammation in post-COVID-19 populations. For example, Tartibian et al. observed significant declines in CRP and D-dimer after a structured exercise intervention in COVID-19 survivors, supporting the anti-inflammatory effects of rehabilitation-based recovery protocols [34]. Similarly, Rao et al. reported significant post-rehabilitation reductions in both markers in patients with long COVID [35]. However, given the short-term nature of our intervention and the absence of follow-up data, it remains uncertain whether these biomarker improvements reflect transient responses to increased physical activity or sustained physiological recovery. Longitudinal studies are needed to determine the durability and prognostic value of these changes.

Transient lymphocytopenia is a common manifestation of COVID-19. Wang et al. demonstrated that patients recovering from COVID-19 experience significant alterations in lymphocyte subpopulations, including reductions in T cells, B cells, and natural killer (NK) cells. These changes may contribute to temporary immunosuppression and increased susceptibility to secondary infections, even after clinical recovery [36]. In our study, a reduced WBC count was observed prior to the rehabilitation program, whereas a significant increase in WBCs was noted after its completion. A further analysis revealed sex-related differences: in men, the rise in leukocyte count after rehabilitation was statistically significant (from 9.44 to 12.06 × 10^3^/µL; *p* < 0.05), while in women, no statistically significant changes were observed. Additionally, a negative correlation was found between BMI and changes in WBCs (R = −0.316, *p* < 0.01), suggesting that a higher BMI may weaken the immune response to rehabilitation.

In our study, the respiratory muscle training program effectively improved quality of life and blood gas parameters, suggesting an enhanced exercise tolerance. The intervention was well tolerated and consistently followed by participants who achieved clinically meaningful outcomes through structured, therapist-guided sessions. To the best of our knowledge, this is one of the first studies to evaluate the effectiveness of such a program specifically in hospitalized patients recovering from COVID-19.

A significant strength of our study is its comprehensive approach to evaluating the effectiveness of a structured pulmonary rehabilitation program in a real-world clinical setting. Unlike previous studies on outpatient or home-based interventions, our study assessed hospitalized post-COVID-19 patients, providing valuable insights into this understudied population. The multidisciplinary intervention included supervised respiratory muscle training, aerobic and strength exercises, and motivational breathing therapy, which reflects a holistic rehabilitation model aligned with international guidelines. Furthermore, a wide range of outcomes was assessed, including objective measures of pulmonary function (spirometry, plethysmography, gasometry), inflammatory markers (CRP, D-dimer, WBC), and validated quality-of-life domains (WHOQOL-BREF), allowing for a multidimensional evaluation of treatment effects. High adherence to the intervention and standardized testing protocols enhanced the internal validity of our findings.

Undoubtedly, our study has certain limitations that affect the evaluation of the rehabilitation program’s effectiveness. Despite the sample size being calculated in advance, the limited scale of this study may reduce its statistical power and generalizability. Multicenter studies with larger cohorts could provide more representative and objective data. Moreover, our study did not include the standard six-minute walk test (6MWT), which may hinder direct comparison with previous research. Additionally, the intervention’s medium- and long-term effects were not assessed, as the primary aim was to evaluate rehabilitation efficacy in an evolving clinical challenge. What is more, potential behavioral changes such as smoking reduction or weight loss were not objectively assessed during this study, which limits our ability to determine their contribution to the observed improvements in inflammatory markers and quality of life. An additional limitation of this study is its non-randomized design and the absence of a control group of COVID-19 patients who did not undergo rehabilitation, which limits the ability to infer causality. All participants were enrolled as part of a structured post-COVID care program, and withholding intervention was neither feasible nor ethically appropriate in this clinical setting. However, without a non-intervention comparator, the potential contributions of spontaneous recovery or regression to the mean cannot be ruled out as alternative explanations for the observed improvements. While our findings suggest clinically meaningful changes in pulmonary function, inflammatory markers, and quality of life, future randomized or controlled cohort studies are needed to isolate the specific effects of pulmonary rehabilitation. Additionally, comparing outcomes with idiopathic pulmonary fibrosis (IPF), as performed in the study by Gloeckl et al. [6], could have provided valuable context, especially given the differing pathophysiology: COVID-19 typically results in acute lung injury, whereas IPF is a chronic, progressive disease.

Future research should focus on conducting large-scale, multicenter randomized controlled trials to confirm the effectiveness of comprehensive pulmonary rehabilitation in diverse populations of post-COVID-19 patients. Long-term follow-up is essential to determine the sustainability of the observed benefits in pulmonary function, inflammatory markers, and quality of life. Additionally, comparative studies examining different modes of rehabilitation delivery, such as inpatient, outpatient, and telerehabilitation, may help identify the most effective and accessible approaches for various patient subgroups. Given the observed sex-specific differences in rehabilitation outcomes and the potential influence of BMI and inflammatory status, future studies should also explore personalized rehabilitation strategies that consider individual patient characteristics. Finally, incorporating functional performance tests such as the 6MWT and integrating psychological assessments may provide a more comprehensive understanding of the impact of rehabilitation.

## 5. Conclusions

This study demonstrates that a comprehensive pulmonary rehabilitation program is feasible and effective in improving pulmonary function, reducing systemic inflammation, and enhancing quality of life in hospitalized patients with post-COVID-19 syndrome. Significant improvements were observed across spirometric, plethysmographic, and blood gas parameters, as well as in physical and psychological domains of quality of life. The program was well tolerated and highly adhered to, with clinically meaningful benefits achieved through therapist-guided respiratory muscle training and physical activity. These findings highlight the value of early, structured rehabilitation in the recovery process of post-COVID-19 patients and support its integration into standard post-acute care pathways. Further research is warranted to evaluate long-term outcomes and optimize individualized rehabilitation protocols.

## Figures and Tables

**Table 1:** Demographic characteristics of participants.

		N	%	Female		Male		
Gender	Female	30	39	N	%	N	%	
	Male	47	61					
Residence	City/town	49	63.6	21	70	28	59.6	χ^2^ = 0.86; p(χ^2^) > 0.05
	Countryside	28	36.4	9	30	19	40.4	
Education	Primary	1	1.3	0	0	1	2.1	χ^2^ = 3.579; p(χ^2^) > 0.05
	Vocational	16	20.8	6	20	10	21.3	
	Middle	33	42.9	10	33.3	23	48.9	
	High	27	35.1	14	46.7	13	27.7	
Work	White collar	22	28.6	13	43.3	9	19.1	F = 5.138; p(F) > 0.05
	Blue collar	47	61.0	15	50	32	68.1	
	Mixed white and blue collar	8	10.4	2	6.7	6	12.8	
Body mass category	Normal	13	16.9	6	20	7	14.9	**χ^2^ = 8.585;** **p(χ^2^) < 0.05**
	Overweight	41	53.2	10	33.3	31	66	
	Obesity	23	29.9	14	46.7	9	19.1	
Smoking cigarettes	Yes, regularly	24	31.2	6	20	18	38.3	**F = 7.261;** **p(F) < 0.05**
	Yes, occasionally	5	6.5	0	0	5	10.6	
	No	48	62.3	24	80	24	51.1	

**Table 2:** Results in primary and secondary outcomes showing the effect of the pulmonary rehabilitation in people with post-COVID condition.

Parameter	Before Rehabilitation							After Rehabilitation							Wilcoxon’s Test
	M	SD	Me	Min	Max	Q1	Q3	M	SD	Me	Min	Max	Q1	Q3	
**Pulmonary function**															
FVC %norm	73.43	12.09	75.00	50.90	102.30	67.00	81.10	79.14	10.63	78.40	56.00	106.00	71.00	88.00	**Z = −7.386;** ***p* < 0.001**
FEV1%norm	78.91	12.87	78.20	48.20	109.30	70.00	88.20	83.97	11.31	80.50	52.00	112.30	77.30	90.70	**Z = −6.386;** ***p* < 0.001**
FEV1/FVC%norm	101.51	8.25	102.0	77.70	123.30	98.00	106.90	103.14	13.00	103.70	11.90	123.30	99.50	109.00	**Z = −5.367;** ***p* < 0.001**
PEF%norm	80.52	17.78	78.70	37.20	143.00	67.00	89.00	82.60	15.98	81.00	49.00	137.00	73.00	90.00	**Z = −4.468;** ***p* < 0.001**
MEF75%norm	75.72	17.85	75.00	40.60	133.00	65.00	83.40	76.92	16.23	77.00	46.00	123.40	67.00	85.00	**Z = −4.599;** ***p* < 0.001**
MEF50%norm	71.69	15.96	70.50	21.40	123.70	64.00	77.40	74.08	15.31	71.80	38.00	120.50	65.70	81.00	**Z = −5.449;** ***p* < 0.001**
MEF25%norm	65.50	15.04	65.80	27.30	102.00	55.00	73.00	68.44	16.49	67.00	32.00	136.00	56.70	76.40	**Z = −5.601;** ***p* < 0.001**
TLC%norm	67.31	11.63	67.30	42.00	101.00	58.90	73.00	77.70	51.23	71.00	6.00	505.90	66.40	79.00	**Z = −6.416;** ***p* < 0.001**
DLCO%norm	66.30	11.69	67.80	38.00	89.00	59.50	75.00	78.70	56.62	74.00	49.00	561.50	65.90	78.00	**Z = −6.842;** ***p* < 0.001**
**Inflammatory markers**															
CRP (mg/dL)	8.41	6.05	8.90	0.09	27.40	3.40	11.30	2.32	2.35	1.32	0.03	9.50	0.62	3.13	**Z = −7.525;** ***p* < 0.001**
D-Dimer (ng/mL)	2273.00	2554.91	1722.20	98.50	15,889.70	876.60	2464.30	492.99	1017.91	203.44	11.70	8597.20	115.00	453.30	**Z = −7.451;** ***p* < 0.001**
WBC × 10^3^/uL	10.57	4.63	9.50	3.49	21.20	6.78	14.29	12.09	6.76	10.01	2.75	27.33	6.84	17.23	Z = −1.765; p > 0.05
**Arterial blood gases**															
PaCO_2_ (mmHg)	35.38	5.08	35.00	21.20	47.30	33.00	38.90	38.35	3.52	38.0	24.9	49.00	36.60	40.00	**Z = −5.174;** ***p* < 0.001**
PaO_2_ (mmHg)	58.35	11.85	59.40	32.00	89.00	52.60	66.00	76.92	6.65	67.00	48.70	87.00	65.00	71.30	**Z = −6.441;** ***p* < 0.001**
**Quality-of-life domains**															
Physical	41.8	11.2	41.0	13.0	69.0	34.5	50.0	56.1	7.9	56.0	31.0	75.0	50.0	63.0	**Z = −6.906;** ***p* < 0.001**
Psychological	53.2	11.7	56.0	19.0	75.0	44.0	63.0	59.9	8.2	56.0	44.0	81.0	56.0	69.0	**Z = −4.669;** ***p* < 0.001**
Social relationships	64	20.4	69.0	19.0	94.0	50.0	81.0	65.5	19.5	69.0	25.0	94.0	50.0	81.0	Z = −1.656; p > 0.05
Environmental	55.6	13.9	56.0	19.0	81.0	44.0	63.0	64.8	11.5	63.0	38.0	94.0	56.0	75.0	**Z = −5.909;** ***p* < 0.001**

**Table 3:** Linear regression analysis for correlation between WHO-QOL-BREF domains and selected results to assess rehabilitation effect.

Effect	Factor	Unstandardized Coefficients		Standardized Coefficients	t	*p*
		B	Std. Error	Beta		
Rehabilitation effect— physical domain	no factors in the model					
Rehabilitation effect— psychological domain	Gender	−8.43	2.30	−0.40	−3.66	**<0.001**
Rehabilitation effect— social relationships domain	CRP (mg/dL)	1.23	0.34	0.42	3.64	**<0.001**
	d-dimer (ng/mL)	0.00	0.00	−0.42	−3.60	**<0.001**
Rehabilitation effect— environmental domain	Gender	−8.00	2.26	−0.39	−3.53	**<0.001**

## Data Availability

Dataset available on request from the authors.

## Supplementary Materials

The following supporting information can be downloaded at: https://www.mdpi.com/article/10.3390/jcm14134533/s1, Table S1: Results in primary and secondary outcomes divided by gender, showing the effect of the pulmonary rehabilitation in people with post-COVID condition.; Table S2: Results of Mann–Whitney’s test to assess gender impact on analyzed parameters; Table S3: Results of Spearman’s test to assess age and BMI impact on analyzed parameters.

## Abbreviations

The following abbreviations are used in this manuscript:

COVID-19	Coronavirus Disease 2019
CRP	C-reactive Protein
DLCO	Diffusing Capacity of the Lung for Carbon Monoxide
FEV1	Forced Expiratory Volume in One Second
FVC	Forced Vital Capacity
MEF	Maximum Expiratory Flow
PaCO_2_	Partial Pressure of Carbon Dioxide
PaO_2_	Partial Pressure of Oxygen
PEF	Peak Expiratory Flow
RMT	Respiratory Muscle Training
SF-36	Short Form (36) Health Survey
TLC	Total Lung Capacity
WBC	White Blood Cells
WHOQOL-BREF	World Health Organization Quality of Life—BREF questionnaire
