# Supplementary material for: Breathe Better After COVID: The Impact of a Two-Week Pulmonary Rehabilitation Program on Pulmonary Function, Inflammatory Markers, and Quality of Life in Post-COVID Syndrome
Source: J Clin Med. 2025 Jun 26;14(13):4533. doi: 10.3390/jcm14134533 (PMC12250514; doi:10.3390/jcm14134533)
Supplement: Supplementary file 1 [file jcm-14-04533-s001.zip › jcm-3678916-supplementary.pdf]

Supplementary Table S1. Results in primary and secondary outcomes divided by gender, showing the effect of the pulmonary rehabilitation in people with post-COVID condition.

Female	Before rehabiltation							After rehabiltation							Wilcoxon's test
	M	SD	Me	Min	Max	Q1	Q3	M	SD	Me	Min	Max	Q1	Q3	
Pulmonary function															
FVC %norm	74,74	13,37	76,15	50,90	102,30	67,00	86,00	79,92	11,16	79,10	59,00	106,00	71,00	88,30	**Z=-4,628; p < 0,001**
FEV1%norm	79,84	13,69	78,50	57,00	109,30	67,00	89,40	82,47	11,56	79,80	66,00	110,40	77,00	89,90	**Z=-3,007; p < 0,01**
FEV1/FVC%norm	102,56	8,29	102,95	82,0	120,5	98,3	108,0	100,65	18,42	103,4	11,9	121,0	99,7	108,8	**Z=-2,756; p < 0,01**
PEF%norm	83,30	19,01	78,00	54,00	143,00	70,30	89,30	84,07	14,38	87,00	54,70	117,00	77,00	90,70	**Z=-2,892; p < 0,01**
MEF75%norm	80,16	18,79	77,00	44,00	133,00	67,00	89,00	78,53	16,23	77,90	46,00	118,00	69,00	90,00	**Z=-3,009; p < 0,001**
MEF50%norm	76,96	16,32	76,00	45,00	123,70	66,00	87,00	76,32	14,94	77,00	46,00	112,00	66,00	87,90	**Z=-3,116; p < 0,001**
MEF25%norm	68,54	14,33	67,15	43,00	102,00	58,50	76,00	68,70	14,11	68,00	44,00	101,00	57,00	76,00	**Z=-2,949; p < 0,001**
TLC%norm	66,12	9,83	67,00	46,00	84,00	59,00	72,00	69,72	15,90	69,00	6,00	110,00	63,00	77,00	**Z=-4,003; p < 0,001**
DLCO%norm	67,13	12,47	71,00	45,00	88,00	59,00	75,40	72,86	10,36	76,00	57,50	89,00	62,00	78,80	**Z=-4,519; p < 0,001**
Inflammatory markers															
CRP (mg/dl)	7,37	5,69	7,34	0,09	18,80	1,40	10,67	2,57	2,45	2,12	0,03	9,50	0,68	3,13	**Z=-4,638; p < 0,001**
D-Dimer (ng/ml)	2422,7	2896,4	1722,8	211,3	15889,7	1023	2464,3	403,4	425,84	213,2	106	1472,5	133,5	437,6	**Z=-4,7; p < 0,001**
WBC x10*3/ul	12,29	4,44	12,39	4,45	19,37	8,55	15,70	12,13	7,15	8,84	4,12	27,10	5,67	18,30	Z=-0,043; p > 0,05
Arterial blood gases															
$PaCO_2$ (mmHg)	34,80	4,73	34,00	26,20	47,30	33,00	37,30	38,34	3,02	38,05	33,00	46,90	36,00	40,00	**Z=-3,137; p < 0,001**
PaO_2 (mmHg)	58,66	11,19	58,80	36,00	74,90	54,30	70,00	68,97	6,36	67,00	61,20	81,00	64,50	72,00	**Z=-4,249; p < 0,001**
Quality of life domains															
Physical	40,17	7,13	41,00	25,00	56,00	38,00	44,00	56,53	8,01	56,00	38,00	75,00	50,00	63,00	**Z=-4,803; p < 0,001**

Psychological	49,53	13,41	47,00	19,00	69,00	44,00	63,00	60,73	7,32	63,00	50,00	81,00	56,00	63,00	**Z=-4,122; p < 0,001**
Social relationships	56,50	21,77	50,00	19,00	94,00	44,00	75,00	61,47	22,08	56,00	25,00	94,00	50,00	75,00	**Z=-2,548; p < 0,05**
Environmental	49,17	14,22	50,00	19,00	69,00	44,00	63,00	62,43	11,51	63,00	38,00	81,00	56,00	69,00	**Z=-4,564; p < 0,001**

**Male**	**Before rehabiltation**							**After rehabilitation**							**Wilcoxon's test**
	**M**	**SD**	**Me**	**Min**	**Max**	**Q1**	**Q3**	**M**	**SD**	**Me**	**Min**	**Max**	**Q1**	**Q3**	
Pulmonary function															
FVC %norm	72,59	11,27	73,00	52,00	97,50	65,80	78,00	78,63	10,37	78,30	56,00	98,50	71,00	83,00	**Z=-5,784; p < 0,001**
FEV1%norm	78,31	12,42	78,00	48,20	109,20	70,00	88,00	84,93	11,17	84,60	52,00	112,30	77,80	90,90	**Z=-5,515; p < 0,001**
FEV1/FVC%norm	100,84	8,25	101,90	77,70	123,30	97,60	106,50	104,73	7,68	104,00	89,00	123,30	99,20	109,70	**Z=-4,678; p < 0,001**
PEF%norm	78,75	16,92	79,00	37,20	121,00	66,00	88,00	81,67	17,00	80,50	49,00	137,00	67,50	89,30	**Z=-3,416; p < 0,001**
MEF75%norm	72,88	16,82	75,00	40,60	119,00	60,00	80,00	75,89	16,32	76,30	47,00	123,40	66,30	81,00	**Z=-3,502; p < 0,001**
MEF50%norm	68,33	14,95	70,00	21,40	110,20	60,00	76,00	72,64	15,53	70,90	38,00	120,50	65,00	79,00	**Z=-4,493; p < 0,001**
MEF25%norm	63,55	15,30	65,00	27,30	98,00	54,00	73,00	68,28	18,00	66,00	32,00	136,00	56,00	77,00	**Z=-4,842; p < 0,001**
TLC%norm	68,07	12,68	67,90	42,00	101,00	57,90	77,00	82,79	64,10	72,00	55,30	505,90	67,00	79,50	**Z=-5,061; p < 0,001**
DLCO%norm	65,76	11,27	67,00	38,00	89,00	59,50	73,50	82,43	72,06	72,50	49,00	561,50	67,00	77,70	**Z=-5,202; p < 0,001**
Inflammatory markers															
CRP (mg/dl)	9,07	6,24	10,25	0,32	27,40	3,40	11,60	2,16	2,29	1,28	0,12	9,01	0,60	3,13	**Z=-5,947; p < 0,001**
D-Dimer (ng/ml)	2177,4	2339,1	1578	98,5	11021	576,8	2478,1	550,1	1260,5	198,6	11,7	8597,2	106,9	491	**Z=-5,81; p < 0,001**
WBC x10*3/ul	9,44	4,44	8,07	3,49	21,20	6,10	12,74	12,06	6,58	10,64	2,75	27,33	7,33	16,70	**Z=-2,294; p < 0,05**
Arterial blood gases															
$PaCO_2$ (mmHg)	35,74	5,30	37,00	21,20	45,00	33,20	39,70	38,36	3,84	38,00	24,90	49,00	36,70	40,00	**Z=-4,006; p < 0,001**
PaO_2 (mmHg)	58,16	12,38	59,40	32,00	89,00	52,12	66,00	82,00	98,76	67,10	48,70	743,00	65,00	71,30	**Z=-4,832; p < 0,001**

Quality of life domains														
Physical	43,00	13,16	41,00	13,00	69,00	31,00	56,00	55,87	7,85	56,00	31,00	69,00	50,00	63,00	**Z=-5,03; p < 0,001**
Psychological	55,60	10,07	56,00	38,00	75,00	44,00	63,00	59,36	8,81	56,00	44,00	81,00	56,00	69,00	**Z=-2,347; p < 0,05**
Social relationships	68,81	18,20	69,00	25,00	94,00	56,00	81,00	68,11	17,41	69,00	31,00	94,00	56,00	81,00	Z=-0,278; p > 0,05
Environmental	59,87	12,21	56,00	38,00	81,00	50,00	69,00	66,36	11,32	63,00	50,00	94,00	56,00	75,00	**Z=-3,603; p < 0,001**

Supplementary Table S2. Results of Mann Whitney's test to assess gender impact on analyzed parameters.

		Sex							Mann Whitney's test
		M	SD	Me	Min	Max	Q1	Q3	
Pulmonary function									
FVC %norm	Female	5,19	7,35	3,75	-1,60	40,00	1,70	6,00	Z=-0,564; p > 0,05
	Male	6,04	7,98	4,00	-2,90	39,00	1,90	6,10	
FEV1%norm	Female	2,63	5,75	1,25	-10,40	26,00	0,40	5,00	**Z=-3,12; p < 0,01**
	Male	6,62	8,14	4,00	-8,20	38,00	2,00	8,50	
FEV1/FVC%norm	Female	-1,91	18,44	1,20	-98,60	6,30	0,40	2,90	Z=-1,891; p > 0,05
	Male	3,90	5,93	2,00	-5,50	22,70	0,70	6,00	
PEF%norm	Female	0,77	8,78	1,20	-42,50	11,00	0,70	3,00	Z=-0,173; p > 0,05
	Male	2,92	14,12	1,30	-47,00	71,00	0,30	2,50	
MEF75%norm	Female	-1,63	16,22	1,15	-87,00	5,70	0,60	1,80	Z=-0,283; p > 0,05
	Male	3,00	13,14	1,00	-30,00	63,40	0,00	2,00	
MEF50%norm	Female	-0,64	14,53	1,00	-74,70	19,60	0,50	1,70	Z=-1,174; p > 0,05
	Male	4,31	11,86	1,00	-15,00	66,50	0,60	3,00	
MEF25%norm	Female	0,16	10,69	1,00	-51,00	21,00	0,70	1,90	Z=-1,34; p > 0,05
	Male	4,73	9,63	1,50	-8,10	51,00	0,90	5,90	
TLC%norm	Female	3,60	13,55	3,65	-61,00	26,00	2,00	7,00	Z=-0,408; p > 0,05
	Male	14,72	66,52	4,00	-22,00	455,00	1,30	7,50	
DLCO%norm	Female	5,73	5,61	4,75	-4,00	27,20	2,50	7,00	Z=-0,157; p > 0,05
	Male	16,66	72,84	4,00	-12,00	502,50	2,00	8,10	
Inflammatory markers									
CRP (mg/dl)	Female	-4,80	4,08	-4,31	-14,50	1,00	-7,97	-1,07	Z=-1,546; p > 0,05
	Male	-6,90	5,64	-6,36	-26,10	0,13	-10,26	-1,94	
D-Dimer (ng/ml)	Female	-2019,33	2778,81	-1401,25	-15282,70	323,30	-2069,99	-762,40	Z=-0,93; p > 0,05
	Male	-1627,26	1837,10	-1206,22	-9864,00	535,20	-2068,10	-389,10	
WBC x10*3/ul	Female	0,08	6,74	-1,79	-11,15	11,80	-4,55	6,34	

	Male	2,53	7,02	2,71	-11,32	20,08	-2,61	6,25	Z=-1,223; p > 0,05
Arterial blood gases									
$PaCO_2$ (mmHg)	Female	3,54	4,55	3,75	-8,00	11,80	2,00	6,70	Z=-1,458; p > 0,05
	Male	2,61	4,09	2,80	-8,00	12,30	1,00	4,80	
PaO_2 (mmHg)	Female	10,31	10,65	8,70	-7,10	41,10	3,70	13,00	Z=-0,543; p > 0,05
	Male	23,84	102,20	7,60	-20,00	704,40	2,10	17,20	

Supplementary Table S3. Results of Spearman's test to assess age and BMI impact on analyzed parameters.

Spearman's rank correlation coefficient		Age	BMI
Pulmonary function			
FVC%norm	R	-0,209	0,030
	p	0,0677	0,7943
	N	77	77
**FEV1%norm**	**R**	**-0,271**	-0,039
	**p**	**0,0173**	0,7363
	**N**	**77**	77
FEV1/FVC%norm	R	-0,075	-0,148
	p	0,5148	0,1975
	N	77	77
PEF%norm	R	0,136	0,013
	p	0,2382	0,9109
	N	77	77
MEF75%norm	R	0,173	0,041
	p	0,1322	0,7255
	N	77	77
MEF50%norm	R	-0,112	0,099
	p	0,3330	0,3898
	N	77	77
MEF25%norm	R	-0,040	0,125
	p	0,7282	0,2791
	N	77	77

TLC%norm	R	0,130	0,024
	p	0,2596	0,8382
	N	77	77
DLCO%norm	R	0,125	0,087
	p	0,2794	0,4531
	N	77	77
Inflammatory markers			
CRP (mg/dl)	R	0,132	-0,082
	p	0,2541	0,4780
	N	77	77
D-Dimer (ng/ml)	R	0,102	0,017
	p	0,3777	0,8837
	N	77	77
**WBC x10*3/ul**	**R**	0,122	**-0,316**
	**p**	0,3031	**0,0065**
	**N**	73	**73**
Arterial blood gases			
$PaCO_2$ (mmHg)	R	0,222	-0,144
	p	0,0521	0,2123
	N	77	77
PaO_2 (mmHg)	R	0,064	0,037
	p	0,5797	0,7490
	N	77	77
